# Draft Genome Sequence of Escherichia coli KL53

**DOI:** 10.1128/genomeA.00220-18

**Published:** 2018-03-29

**Authors:** Katarina Soltys, Silvia Vavrova, Jaroslav Budis, Lenka Palkova, Gabriel Minarik, Jozef Grones

**Affiliations:** a Faculty of Natural Sciences, Department of Molecular Biology in Bratislava, Comenius University in Bratislava, Bratislava, Slovakia; b Faculty of Mathematics, Physics and Informatics in Bratislava, Department of Computer Science Bratislava, Comenius University in Bratislava, Bratislava, Slovakia; c Faculty of Medicine, Institute of Molecular Biomedicine, Comenius University in Bratislava, Bratislava, Slovakia; d Geneton Ltd., Bratislava, Slovakia

## Abstract

Here, we report the draft genome sequence of a clinical isolate of the uropathogenic strain Escherichia coli KL53. A total of 5,083,632 bp was *de novo* assembled into 170 contigs containing 89 RNAs and 5,034 protein-coding genes. Remarkable is the presence of the tellurite resistance (*ter*) operon on a plasmid.

## GENOME ANNOUNCEMENT

The genus Escherichia belongs to the family of Enterobacteriaceae, and due to the cooccurrence of antibiotic and heavy metal resistance genes, these organisms can, through plasmid carryover, evolve multidrug and multimetal resistance, including tellurium resistance. Tellurite oxyanions are highly toxic for most forms of life, even at micromolar levels. A group of pathogens, including foodborne pathogen Escherichia coli O157:H7, Klebsiella pneumoniae (1), Bacillus subtilis, Staphylococcus aureus, Yersinia pestis (2), Vibrio cholerae, and Shigella spp., possess tellurite resistance genes; E. coli KL53 has this cluster on a plasmid (3).

We report here the draft genome sequence of a uropathogenic isolate of E. coli strain KL53 serotype O18:H14 recovered from the hospital environment of the Department of Urology of University Hospital in Bratislava, Slovakia. The strain was found to grow at a high concentration of tellurite under laboratory conditions. The complete genome was sequenced by next-generation sequencing. The sequencing libraries were prepared using the Tn-based library preparation chemistry Nextera XT (Illumina, Inc., San Diego, CA, USA), according to the manufacturer’s instructions. One nanogram of total DNA was fragmented and amplified. Purified fragments were quantified fluorometrically with a Qubit fluorimeter (Thermo Fisher Scientific, Waltham, MA, USA). The quality was assessed using a high-sensitivity (HS) DNA chip (Agilent Technologies, Waldbronn, Germany). Paired-end sequencing was performed using an Illumina MiSeq platform (Illumina, Inc.). The sequence quality was checked with the bioinformatics tool FastQC. For the *de novo* assembly, SPAdes version 3.10.1 was used. Gene identification and annotation were provided by the NCBI Prokaryotic Genome Annotation Pipeline (https://www.ncbi.nlm.nih.gov/genome/annotation_prok/), with the best-placed reference set and GeneMarkS+ version 4.2.

The total sequence length was 5,083,632 bp. *De novo* assembly of the E. coli KL53 genome resulted in a set of 170 contigs with GC content of 50.77%. The total DNA contains 5,418 genes, of which 5,034 genes were protein-coding and 89 were RNA genes (14 rRNA, 66 tRNA, and 9 noncoding RNA [ncRNA]). The presence of 3 plasmid incompatibility group-determining sequences was detected [IncI1, 100% identity; IncHI2, 100% identity; IncFIB(K), 98.93% identity] by PlasmidFinder (https://cge.cbs.dtu.dk/services/PlasmidFinder/). An exact match of IncI1 plasmid genes *repl1-ardA-trbA-sogS-pilL* with database alleles 1-4-17-1-3 was determined using pMLST (http://pubmlst.org/plasmid/).

The KL53 strain includes multiple heavy metal resistance genes for zinc (*znuC*, *znuB*, *zupT*, and *zntB*), copper (*copA* to *copD*), a cation efflux system for silver and other heavy metals (*cusA*, *cusC*, and *cusF*), arsenic (*arsCBR*), and a tellurium resistance system (*terZABCDEF* and *terWY1XY2Y3*), part of which was determined to be localized on a plasmid by transconjugation (GenBank accession no. AJ888883) (4).

Multidrug resistance genes include multidrug efflux resistance-nodulation-division (RND) transporter MexE, which is involved in resistance to a number of compounds, including lipophilic antibiotics, multidrug and toxic compound extrusion (MATE) family efflux transporter DinF, Bcr/CflA (bicyclomycin resistance protein), and the BlaEC family class C β-lactamase providing multiresistance to β-lactam antibiotics.

Furthermore, genes for the resistance to bacitracin, UV resistance, and resistance to N4 and lambda phages were detected in annotated draft genome sequence of E. coli KL53.

### Accession number(s).

This whole-genome shotgun project has been deposited in GenBank under the accession no. NXEL00000000. This version of the project (01) has the accession no. NXEL01000000 and consists of sequences NXEL01000001 to NXEL01000170.
